# A new species of dactyloid anole (Iguanidae, Polychrotinae, *Anolis*) from the southeastern slopes of the Andes of Ecuador

**DOI:** 10.3897/zookeys.53.456

**Published:** 2010-08-27

**Authors:** Fernando P. Ayala-Varela, Torres-Carvajal Omar

**Affiliations:** Escuela de Biología, Pontificia Universidad Católica del Ecuador, Avenida 12 de Octubre y Roca, Apartado 17-01-2184, Quito, Ecuador.

**Keywords:** Andes, Anolis, Ecuador, new species, Parque Nacional Podocarpus, Polychrotinae, systematics

## Abstract

We describe a new species of Anolis from the southeastern slopes of the Andes of Ecuador, province of Zamora-Chinchipe, Parque Nacional Podocarpus. It belongs to (1) the aequatorialis species-group by being of moderate to large size with narrow toe lamellae, and (2) the eulaemus sub-group by having a typical Anolis digit, in which the distal lamellae of phalanx II distinctly overlap the proximal scales of phalanx I. The new species is most similar morphologically to Anolis fitchi but differs from it mainly by having a dewlap with longitudinal rows of 2−5 granular, minute scales separated by naked skin (longitudinal rows of one or two keeled, large scales separated by naked skin in Anolis fitchi) and a vertically shorter dewlap (longer dewlap in Anolis fitchi).

## Introduction

The lizard genus Anolis (anoles) is the most species-rich genus of amniotes, with nearly 400 described forms (Poe 2004; Nicholson 2002). Although the phylogenetic relationships of many Caribbean and Central American species have been analyzed (e.g., Poe 1998, 2004; Creer et al. 2001; Schneider et al. 2001; Jackman et al. 2002; Nicholson 2002), the relationships of South American species formerly called “Dactyloa” (sensu Guyer and Savage 1986; latifrons series sensu Etheridge 1959) are relatively understudied. Accurate estimation of the relationships of this group and the entire Anolis clade requires taxonomic knowledge of South American species, many of which remain undescribed. Here we contribute to this growing body of taxonomic knowledge (e.g., Ayala-Varela 2004; Poe and Yañez-Miranda 2008; Ugueto et al. 2007) with the description of a new species of Anolis from Ecuador.

During revisionary work on anoles of Ecuador, we examined some specimens of Anolis similar to Anolis fitchi Williams and Duellman 1984 collected at the Parque Nacional Podocarpus in southeastern Ecuador. We found that the color pattern of these specimens differed dramatically from typical Anolis fitchi. Detailed examination of these specimens revealed other differences in squamation and color pattern indicative of separate species status.

## Materials and methods

Specimens examined (Appendix and description below) are housed in the herpetological collections of the Escuela Politécnica Nacional, Quito (EPN), Fundación Herpetológica Gustavo-Orcés, Quito (FHGO), Museum of Comparative Zoology, Harvard University (MCZ), Museo Ecuatoriano de Ciencias Naturales, Quito (MECN) and Museo de Zoología, Pontificia Universidad Católica del Ecuador, Quito (QCAZ).

External character terminology follows Williams et al. (1995). Scale counts were made on the left side if applicable. Ten morphological measurements were taken with digital calipers to the nearest 0.1 mm: head length, head width, head height, forelimb length, hindlimb length, snout-vent length (SVL), snout length, ear opening maximum length, interparietal length, and dewlap height. In addition, tail length was measured with a ruler to the nearest 1 mm. Regenerated or broken tails were not measured. Sex was determined by the presence of hemipenes and size of the dewlap. Egg volume was calculated using the formula for the prolate spheroid: V = 4/3 π (length/2) x (width/2)2. Osteological characters were observed in a cleared-and-double stained adult female specimen (QCAZ 6047).

All measurements were used in statistical analyses performed in PAST 1.27 (Hammer et al. 2004). Differences in quantitative characters between the new species and Anolis fitchi were evaluated with t-tests. One of the assumptions of the t-test for two samples is that the variances of both samples are equal: therefore, F-tests also were performed for each character to test for equality of variances. If the variances were not the same (i.e., P < 0.05), an unequal variance t-statistic (Welch test) was used.

The distribution map was prepared in ArcMap 9.3 (ESRI, Inc.); WGS84 is the datum for all coordinates presented below.

## Results

### Anolis podocarpus sp. n.

urn:lsid:zoobank.org:act:BBCF5322-9297-49D2-B910-ACCC374F9890

#### Holotype.

**Figure 1.**
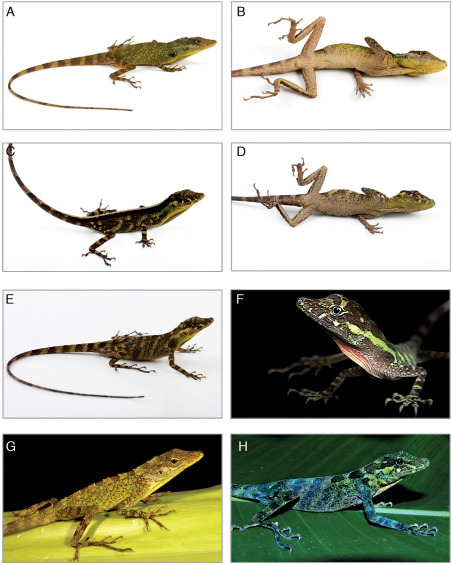
Two species of Anolis from eastern Ecuador. Anolis podocarpus sp. n.: holotype male (**A**, **B**, QCAZ 10126), female (**C**, **D**, QCAZ 10127), female (**E**, QCAZ 10129), juvenile (**F**, QCAZ 6200); Anolis fitchi: male (**G**, QCAZ 8770), female (**H**, QCAZ 9707). Photographs by L.A. Coloma (male of Anolis fitchi), F. Ayala-Varela (juvenile of Anolis podocarpus sp. n. and female of Anolis fitchi) and S.R. Ron (male and females of Anolis podocarpus sp. n.).

QCAZ 10126 (Fig. 1A,B), adult male, Ecuador, Provincia Zamora-Chinchipe, Romerillos Alto, 04°13'35.6"S; 78°56'23.0"W, 1550 m, 18 December 2009, collected by Fernando Ayala, Steven Poe, Levi Gray, and Julian Davis.

#### Paratypes.

ECUADOR: QCAZ 6038, 6045–47, 6188, QCAZ 6199–200, same locality data as holotype, 1545–1618 m, 1 August 2002, collected by Fernando Ayala-V. and David Salazar; QCAZ 10127, same collection data as holotype; FHGO 1726–30, 1735, 1755, upper basin of Río Curintza, 04°09'36"S; 78°58'48"W, 25 March and 3 April 1998, collected by D. Almeida-Reinoso and F. Nogales-Sornosa; FHGO 2406–07, Numbami, upper basin of río Jambué, 04°10'11.9994"S; 78°57'0"W, 1530 m, 30 April 1999, collected by D. Almeida-Reinoso and F. Nogales-Sornosa; EPN 11354, Campamento San Antonio, Refugio de Vida Silvestre El Zarza, Los Encuentros, cantón Yantzaza, 03°50'27.00"S; 78°31'38.92"W, 1556 m, 07 November 2008, collected by A. Almendáriz, Marco Salazar and Marco Angamarca; EPN 11355, same locality data as former, 14 November 2008, collected by A. Almendáriz, Luis Benalcázar, Marco Angamarca and W. Torres.

#### Diagnosis.

The new species belongs to the punctatus-subsection (Williams 1976a) by having an arrow-shaped interclavicle [T-shaped in the carolinensis-subsection Williams 1976a]. Within the punctatus-subsection, Anolis podocarpus is a member of (1) the latifrons-series sensu Etheridge 1959 by having at least four parasternal chevrons attached to the dorsal ribs, and the lateral processes of the interclavicle divergent from the proximal parts of the clavicles; (2) the aequatorialis species-group (Williams 1976b) by being of moderate to large size (SVL = 73.6−96.0 mm), with narrow toe lamellae; and (3) the eulaemus-subgroup (Williams and Duellman 1984) by having a typical Anolis digit, in which the distal lamellae of phalanx II distinctly overlap the first proximal subdigital scale of phalanx I. The new species lacks transverse processes on most or all of the autotomic caudal vertebrae.

**Figure 2.**
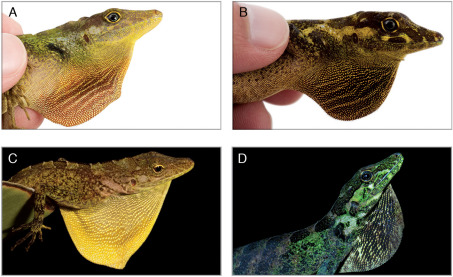
Dewlap of Anolis podocarpus sp. n.(**A**, holotype male, QCAZ 10126; **B**, female, QCAZ 10129) and Anolis fitchi (**C**, male, QCAZ 8770; **D**, female, QCAZ 9707). Photographs by L.A. Coloma (male of Anolis fitchi), F. Ayala-Varela (female of Anolis fitchi) and S.R. Ron (male and female of Anolis podocarpus sp. n.).

**Figure 3.**
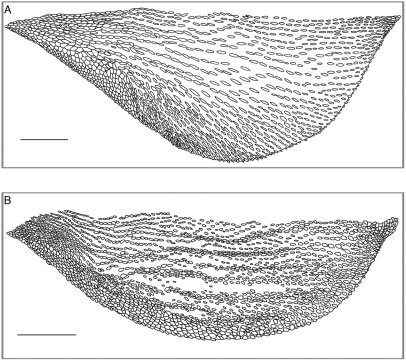
Male dewlap of Anolis fitchi (**A**, QCAZ 9028) and Anolis podocarpus sp. n. (**B**, QCAZ 10126) in lateral view. Scale bar = 5 mm. Illustrations by D. Paucar-Guerrero.

Among species in the eulaemus-subgroup (Anolis antioquiae Williams 1985, Anolis eulaemus Boulenger 1908, Anolis fitchi, Anolis gemmosus O’Shaughnessy 1875,Anolis maculigula Williams 1984, Anolis megalopithecus Rueda-Almonacid 1989 and Anolis ventrimaculatus Boulenger 1911), Anolis podocarpus differs from Anolis antioquiae (character states in parentheses) in lacking a canthal ridge projecting above the loreal region (very sharp canthal ridge projecting above the loreal region), and 8−11 supralabials (6−7). Anolis podocarpus can be distinguished from Anolis gemmosus by having a SVL > 70 mm in adults (SVL < 70 mm in Anolis gemmosus), and from Anolis megalopithecus by having 6−9 postmental scales (3−4 in Anolis megalopithecus). From the remaining species in the eulaemus-subgroup (character states in parentheses), Anolis podocarpus differs by the combination of the following characters: (1) dewlap moderate in size in females (rudimentary in Anolis eulaemus and Anolis maculigula; absent in Anolis gemmosus and Anolis ventrimaculatus); (2) dewlap skin uniform reddish brown or terracotta, with a dark brown tint anteriorly and orange or pink tint posteriorly in males, Fig. 2 (pale brown in Anolis eulaemus; dark brown, with a pale yellowish brown edge in Anolis fitchi; dull yellowish green, or bluish green proximally shading to yellow or orange distally in Anolis gemmosus; bluish gray proximally, with anterior third pale bluish rose and posterior portion white becoming pale blue towards the belly in Anolis maculigula; red in males and sepia in females in Anolis megalopithecus; dark brown or orange covered by yellow rows of scales and a dark blotch at its base in Anolis ventrimaculatus); (3) dewlap skin uniform dark violet, with a brownish-red tint in females (reddish orange, with black blotches and yellow border in Anolis antioquiae; dark brown in Anolis eulaemus; yellowish green to brown, with dark brown blotches in Anolis fitchi; sepia in Anolis megalopithecus); (4) dewlap with longitudinal rows of 2−5 granular, minute scales separated by naked skin, Fig. 3 (longitudinal rows of one or two keeled, large scales separated by naked skin in Anolis fitchi andAnolis ventrimaculatus); (5) iris bluish turquoise (iris gray or dull bluish gray in males and blue-green in females of Ecuadorian populations; blue in males and pale blue in females of Colombian populations (Williams and Duellman 1984) in Anolis fitchi; dark brown in Anolis maculigula; reddish brown in females in Anolis megalopithecus); (6) 1−3 scales between supraorbital semicircles (4−5 in Anolis antioquiae, and 5−6 in Anolis megalopithecus); (7) interparietal scale present (absent in Anolis antioquiae andAnolis megalopithecus).

Among all species in the eulaemus-subgroup Anolis podocarpus is most similar morphologically to Anolis fitchi; in addition to the differences mentioned above, Anolis podocarpus can be distinguished from Anolis fitchi (character states in parentheses, Table 1) by having more scales between second canthals (14–20 and 12–20, respectively; t-test, t = 4.126, P < 0.05); more scales bordering the rostral posteriorly (7–12 and 5–10, respectively; t-test, t = 3.551, P < 0.05); more rows of loreals (9–13 and 6–12, respectively; t-test, t = 7.601, P < 0.05); more postmentals (5–9 and 4–8, respectively; t-test, t = 3.119, P < 0.05); shorter dewlap in males (dewlap height/SVL = 0.10–0.23 and 0.18–0.36, respectively; t-test, t = 4.212, P < 0.05); shorter dewlap in females (dewlap height/SVL = 0.11–0.19 and 0.14–0.23, respectively; t-test, t = 3.165, P < 0.05); and sides of neck with light-blue and pale pink small spots in males (sides of neck with a light yellow irregular stripe, Fig. 1G,H).

**Table 1:** Summary of morphological characters and measurements (mm) of Anolis fitchi and Anolis podocarpus sp. n. from Ecuador. For each quantitative character, the F-value, t-value, and corresponding P-values are given. Range and sample size (N) followed by mean ± standard deviation are given.

Character	Anolis fitchi	Anolis podocarpus	F-value	P	t-value	P
Scales between second canthals	12–20 (37) 14.95 ± 1.88	14–20 (19) 17.11 ± 1.79	1.107	0.843	4.126	<0.05
Postrostrals	5–10 (37) 7.68 ± 1.27	7–12 (19) 8.89 ± 1.10	1.333	0.522	3.551	<0.05
Rows of loreals	6–12 (37) 8.38 ± 1.23	9–13 (19) 10.84 ± 0.96	1.655	0.255	7.601	<0.05
Scales between supraorbital semicircles	1–3 (36) 2.28 ± 0.66	1–3 (19) 1.79 ± 0.63	1.094	0.864	2.650	0.011
Scales between interparietal and semicircles	3–7 (37) 4.46 ± 0.99	2–6 (19) 3.79 ± 1.03	1.089	0.800	2.366	0.022
Supralabials	8–11 (37) 9.05 ± 0.81	8–11 (19) 9.26 ± 0.93	1.313	0.474	0.865	0.391
Postmentals	4–8 (37) 6.00 ± 1.00	5–9 (19) 6.89 ± 1.05	1.099	0.782	3.119	<0.05
Lamellae under phalanges II-III of fourth toe	19–25 (37) 22.19 ± 1.76	20–25 (19) 22.42 ± 1.43	1.524	0.342	0.496	0.622
Head length/SVL	0.24–0.28 (30) 0.26 ± 0.01	0.26–0.28 (12) 0.27 ± 0.01	1.957	0.240	2.748	0.009
Head length/head width	1.61–1.94 (30) 1.79 ± 0.07	1.66–1.86 (12) 1.77 ± 0.07	1.009	0.953	0.784	0.438
Head height/head width	0.75–0.86 (30) 0.81 ± 0.03	0.76–0.87 (12) 0.81 ± 0.03	1.097	0.796	0.379	0.706
Forelimb length/SVL	0.50–0.57 (30) 0.54 ± 0.02	0.47–0.61 (12) 0.52 ± 0.04	4.355	<0.05	1.510	0.155
Hindlimb length/SVL	0.87–1.02 (30) 0.94 ± 0.04	0.82–1.00 (12) 0.90 ± 0.06	2.168	0.094	2.411	0.021
Tail length/SVL	1.84–2.57 (15) 2.33 ± 0.20	1.83–2.48 (10) 2.29 ± 0.19	1.101	0.912	0.563	0.579
Dewlap height/SVL in males	0.18–0.36 (17) 0.27 ± 0.06	0.10–0.23 (5) 0.16 ± 0.05	1.340	0.851	4.212	<0.05
Dewlap height/SVL in females	0.14–0.23 (13) 0.19 ± 0.03	0.11–0.19 (7) 0.14 ± 0.04	1.500	0.517	3.165	<0.05
Maximum SVL males	96	96				
Maximum SVL females	87	89				

#### Description of holotype

**Figure 4.**
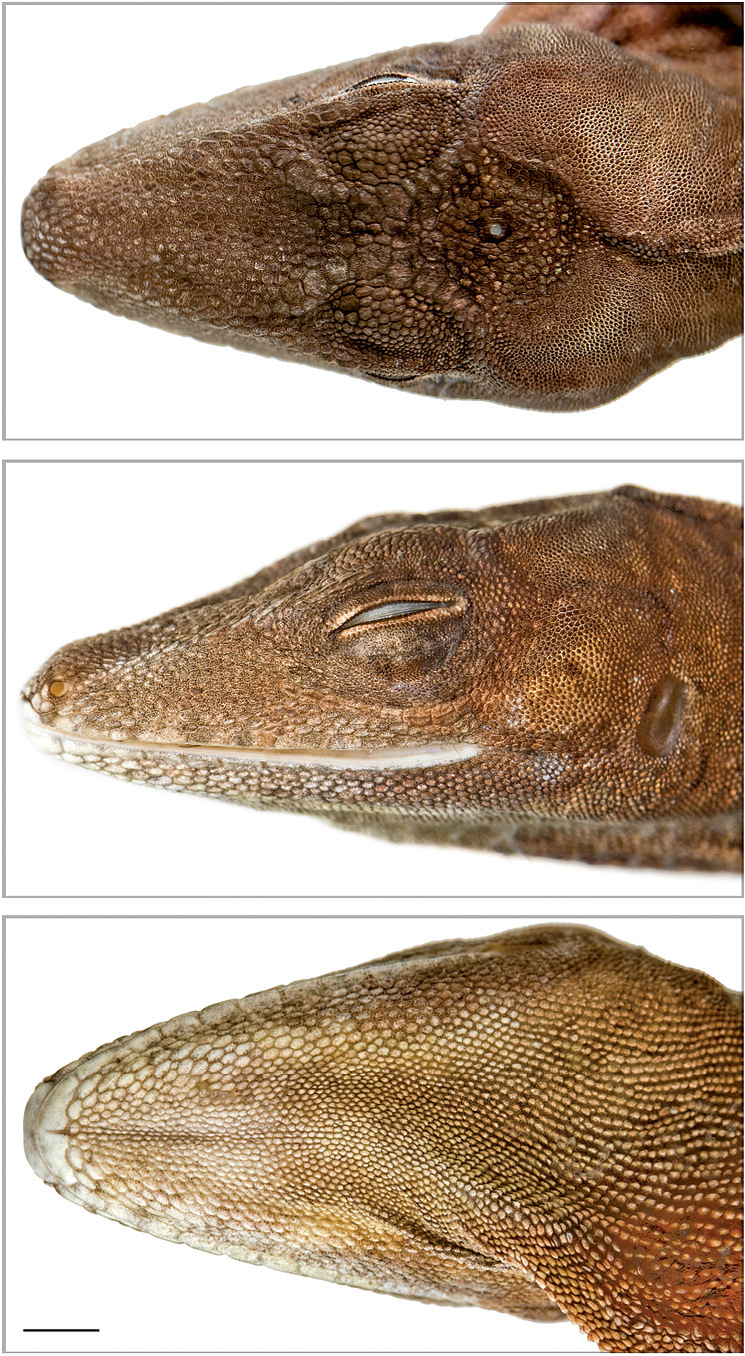
Head of the holotype (QCAZ 10126) of Anolis podocarpus sp. n. in dorsal (top), lateral (middle), and ventral (bottom) views. Photographs by L. Bustamante. Scale bar = 10 mm.

(variation in paratypes in parentheses). Male (Fig. 4);SVL 87.0 mm (73.6−96.0 mm); tail length 192.0 mm (168.0−224.0 mm); head length 23.3 mm (19.3−25.4 mm); head width 13.0 mm (11.5−13.8 mm); head height 11.0 mm (9.0−12.0 mm); forelimb length 44.9 mm (39.3−49.9 mm); hindlimb length 75.4 mm (70.0−83.9 mm); dewlap height 12.4 mm (8.2−20.2 mm); interparietal length 1.1 mm (0.9−2.0 mm); ear opening maximum length 2.72 mm (2.3−2.9 mm); snout length 9.81 mm (8.6−11.6 mm).

Head scales unicarinate (smooth or rugose); 19 (14−20) scales between second canthals; 17 (16−20) scales between first canthals; 10 (7−12) scales bordering the rostral posteriorly; circumnasal separated from rostral by one scale or in contact (anterior nasal or divided anterior nasal in contact with rostral); supraorbital semicircles separated by two (1−3) scales; supraocular disk not differentiated and keeled; one (1–2) short superciliary followed by granules; 12 (9−13) loreal rows; 113 loreal scales (81–135); interparietal smaller (much smaller or similar) than ear opening; 3−5 (2−6) scales between interparietal and semicircles on each side; scales behind interparietal grading into nape scales; suboculars and supralabials separated (in contact) by one scale; 10 (8−11) supralabials counted up to a point below center of eye; 9 (9−11) infralabials counted up to a point below center of eye; six (5−9) postmentals; enlarged sublabials absent (one enlarged sublabial in contact with infralabials).

Dorsal crest absent; two enlarged middorsal rows (enlarged middorsal rows absent); dorsals swollen, unicarinate or conical; flank scales more or less separated by skin (juxtaposed); ventrals equal than dorsals (ventrals larger than dorsals); ventrals slightly protuberant, smooth, and subimbricate (separated from each other by skin or juxtaposed).

Toepads overlap the first phalanx in all toes; 20 (20−25) lamellae under second and third phalanges of fourth toe; supradigitals multicarinate; tail weakly compressed; postanals absent (present or inconspicuous).

Nuchal and dorsal folds weakly developed (folds absent in females); dewlap large in both sexes extending posteriorly behind forelimbs, with longitudinal rows of 3−4 (2−5) granular, minute scales separated by naked skin.

Sexual variation of meristic and morphometric characters in Anolis podocarpus is presented in Table 2.

**Table 2:** Sexual variation in scutellation and measurements (mm) of Anolis podocarpus sp. n. Range and sample size (N) followed by mean ± standard deviation are given.

Character	Males	Females
Scales between second canthals	16–20 (6) 17.33 ± 1.75	14–20 (13) 17.00 ± 1.87
Postrostrals	7–10 (6) 8.50 ± 1.22	8–12 (13) 9.08 ± 1.04
Rows of loreals	9–12 (6) 10.83 ± 0.98	9–13 (13) 10.85 ± 0.99
Scales between supraorbital semicircles	1–2 (6) 1.67 ± 0.52	1–3 (13) 1.85 ± 0.69
Scales between interparietal and semicircles	2–6 (6) 3.67 ± 1.51	3–5 (13) 3.85 ± 0.80
Supralabials	8–11 (6) 9.33 ± 1.21	8–11 (13) 9.23 ± 0.83
Postmentals	5–8 (6) 6.50 ± 1.05	6–9 (13) 7.08 ± 1.04
Lamellae under phalanges II-III of fourth toe	20–24 (6) 22.17 ± 1.33	21–25 (13) 22.54 ± 1.51
Head length/Snout-vent length	0.26–0.28 (5) 0.27 ± 0.01	0.26–0.27 (7) 0.26 ± 0.01
Head length/head width	1.77–1.86 (5) 1.81 ± 0.03	1.66–1.83 (7) 1.73 ± 0.07
Head height/head width	0.81–0.87 (5) 0.83 ± 0.02	0.76–0.83 (7) 0.79 ± 0.03
Forelimb length/snout-vent length	0.49–0.58 (5) 0.52 ± 0.04	0.47–0.61 (7) 0.52 ± 0.05
Hindlimb length/Snout-vent length	0.85–0.98 (5) 0.89 ± 0.05	0.82–1.00 (7) 0.91 ± 0.06
Tail length/Snout-vent length	1.83–2.48 (5) 2.26 ± 0.26	2.19–2.45 (5) 2.32 ± 0.11
Dewlap height/Snout-vent length	0.10–0.23 (5) 0.16 ± 0.05	0.11–0.19 (7) 0.14 ± 0.04
Maximum SVL	96	89

#### Coloration in life of holotype

**Figure 5.**
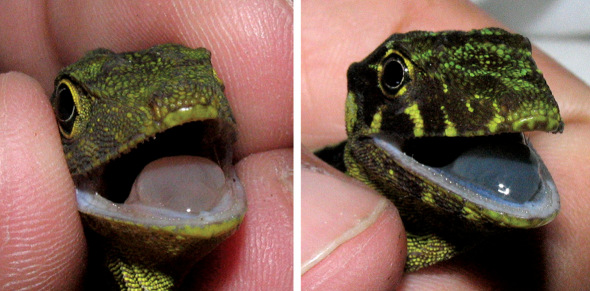
Tongue of Anolis podocarpus sp. n. Left: holotype adult male (QCAZ 10126); right: adult female (QCAZ 10127). Photographs by F. Ayala-Varela.

(Fig. 1A,B). Head, body and limbs green; head with two dark green transverse bands on the supraocular disk separated by one yellowish green transverse band; body with a vertebral series of wide, dark brown blotches that diffuse without reaching flanks; limbs and tail with wide, dark brown transverse bands; side of neck with an aquamarine irregular longitudinal stripe that extends from postocular region above the tympanum to level of shoulder; side of shoulder with a greenish black irregular spot and opaque pink dots; body flanks with small turquoise dots; ventral surface of head pale yellow, with two pairs of lateral, yellow, short bands; ventral surface of body yellowish cream; ventral surface of tail cream anteriorly, with reddish-brown transverse bands posteriorly; ventral surface of hindlimbs pinkish cream with reddish brown reticulations; dewlap skin terracotta with dark brown tint anteriorly and orange tint posteriorly; dewlap scales yellow anteriorly, and white-yellow posteriorly; upper and lower palpebrals yellow; iris bluish-turquoise with white ring; tongue pink (Fig. 5).

#### Coloration in preservative of holotype.

Head, body and limbs brown; head with a light brown transverse band on the supraocular disk; body flanks brown with dark green small reticulations; limbs and tail brown, with wide, dark brown transverse bands; side of neck brown with cream dots; side of shoulder with a black irregular spot and cream dots; ventral surface of head brownish cream; ventral surface of body brownish cream with brown dots; ventral surface of tail cream anteriorly and brown posteriorly; ventral surface of hindlimbs cream with brown reticulations; dewlap skin dark brown; dewlap scales brownish cream with white scales posteriorly; upper and lower palpebrals brownish cream.

#### Color in life variation.

Adult male QCAZ 6038: Head and limbs yellowish green, body green, and tail creamish green; body with a series of seven vertebral wide, yellowish brown bands that diffuse without reaching flanks; sides of head with a light blue irregular stripe extending from postocular region to level of the shoulder; sides of neck with pale pink dots; sides of shoulder with a blackish brown spot and pale pink dots; body flanks green with dark green and turquoise dots, and with dark greenish brown spots assembling alternating bands extending posteroventrally; ventral surface of head greenish yellow anteriorly and pale yellow posteriorly, with two pairs of lateral, yellow, short bands; ventral surface of hindlimbs cream with pale brown reticulations; dewlap skin reddish brown with dark brown tint anteriorly and pink tint posteriorly; dewlap scales greenish yellow anteriorly, and white posteriorly.

**Figure 6.**
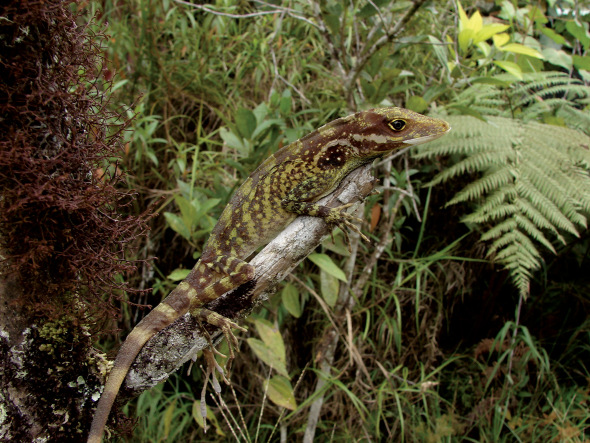
Male of Anolis podocarpus sp. n. (EPN 11355) from El Zarza (Zamora Chinchipe province). Photograph by A. Almendáriz.

Adult male EPN 11355 (Fig. 6) differs from the descriptions above in having a cream irregular stripe that extends from the angle of the jaw above the tympanum to the neck; side of neck with a cream irregular stripe that forms an arc extending from the proximal border of the dewlap to the shoulder; shoulder with a greenish black irregular spot and pink dots.

Adult female QCAZ 10127 (Fig. 1C,D): Head lime-green with two blackish brown transverse bands on supraocular disk; body and tail blackish brown with a whitish-cream vertebral stripe; limbs yellowish green with wide, blackish-brown transverse bands; two subocular pale yellow stripes extending anterodorsally (posteriormost stripe) and posterodorsally (anteriormost stripe) from supralabials; lateral aspect of neck with a longitudinal aquamarine stripe that extends posteriorly from the posterior end of the eye over the tympanum to the level of the forelimb; body flanks yellowish green with dark brown spots that cluster anteriorly, posteriorly, and at midbody; ventral surface of head greenish cream with two pairs of lateral, pale yellow, short bands; ventral surface of body brownish cream with blackish brown spots; ventral surface of limbs brownish cream, with blackish brown reticulations; ventral surface of tail brownish cream on the base, with blackish brown transverse bands; dewlap skin dark violet with a brownish-red tint; dewlap scales yellow; iris bluish-turquoise; tongue dark blue (Fig. 5). Adult female QCAZ 10129 (Fig. 1E) differs from the previous description in having a longitudinal series of five wide, dark brown transverse bands that extend posteroventrally over flanks.

Juvenile QCAZ 6200 (Fig. 1F): Color pattern is similar to adult female QCAZ 10129, but differs in having dewlap skin orange-red with white scales and tongue orange.

#### Natural history and ecology.

An adult female (SVL 89.1 mm, QCAZ 6045) collected on 1 August 2002 and held in captivity until 27 September 2002 laid an egg (18.5 x 8.4 mm; 682.1 mm3) on 24 August 2002. A juvenile (FHGO 1730) was collected on 25 March 1998 (37.0 mm SVL, 82.5 mm tail length); a larger juvenile (QCAZ 6200) was collected on 1 August 2002 (43.5 mm SVL, 96.0 mm tail length).

Specimens of Anolis podocarpus were collected in secondary forest near small streams or in ravines. All individuals were found between 19:00 and 23:00 h sleeping head-up on leaves of ferns, Araceae, Musaceae, on branches 0.30–2.50 m above streams, or on stream shores. This species occurs in sympatry with two undescribed species of anoles at the type locality.

#### Distribution and conservation.

**Figure 7.**
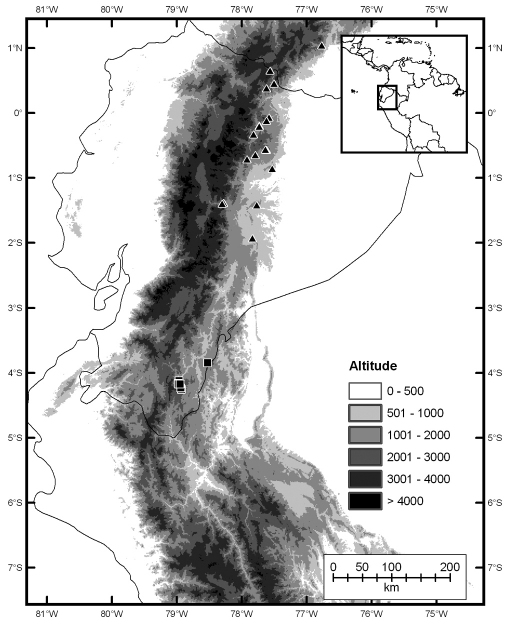
Distribution of Anolis fitchi (triangles) and Anolis podocarpus sp. n. (squares).

Anolis podocarpus inhabits the eastern slopes of the eastern Andean cordillera in southern Ecuador, Zamora-Chinchipe province, between 1530−1910 m (Fig. 7). It is known from the upper basin of the Zamora river (Atlantic drainage) in montane cloud forest and low montane evergreen forest (Sierra 1999). Most individuals of this species have been collected within two protected areas in southern Ecuador, Parque Nacional Podocarpus and Refugio de Vida Silvestre El Zarza, which suggests that at least some populations of Anolis podocarpus are well protected.

#### Etymology.

The specific epithet podocarpus alludes to the conifer Podocarpus and derives from the Greek words pous, podos (=foot), and karpos (=fruit). The tree Podocarpus gives its name to Parque Nacional Podocarpus, where the new species described in this paper was discovered.

## Supplementary Material

XML Treatment for Anolis podocarpus

## Appendix. Additional specimens examined

Anolis fitchi – Ecuador: *Morona-Santiago*: “Chiquara” (sic Chiguaza), 01°55'60S; 77°49'60W, MCZ 124355–56; Cordillera del Cutucú, Comunidad Uuntzuanz, 1250–1300 m, EcoC 1348; *Napo*: Cordillera de los Guacamayos, 2200 m, QCAZ 3112, 3173; El Chaco, 1400 m, QCAZ 3092; Galeras, QCAZ 3758; Parque Nacional Sumaco Napo-Galeras, Cocodrilos, 00°38'57S; 77°47'35W, 1846 m, QCAZ 5439–42; Volcán Sumaco, vertiente Este, 1570–1642 m, QCAZ 920, 926; 30 km N of turnoff to Baeza, 00°17'31.7394S; 77°46'30.6582W 1661 m, QCAZ 9707; *Orellana*: Alto Napo, Loreto, MCZ 124351; Cabecera del río Sola, MCZ 124352; Región de Loreto, MCZ 124350; *Pastaza*: “Río Villaro” (sic Río Villano), 01°25'11.9994S; 77°46'11.9994W, MCZ 124353–54; *Sucumbíos*: El Reventador, Gonzalo Pizarro, EPN 7056–57; El Reventador, Recinto La Libertad, La Virgen, 00°4.29'1S; 77°34.8'1W, 1679 m, QCAZ 5997, 8770; La Bonita, 1500–1590 m, FHGO 446, EPN 3158; La Sofía, 00°22'40N; 77°38'02W, 1648–1800 m, EPN 7583–93; Río Azuela, 1700 m, QCAZ 5425, 5435–38, 5502–03; Santa Bárbara, Sebundoy, 2200 m, EPN 3159; *Tungurahua*: Parque Nacional Los Llanganates, El Topo, 01°23'08S; 78°16'49W, 2150 m, MECN 35; Río Verde, 01°24'16S; 78°17'48.3W, 1512 m, QCAZ 5648–49, 5715.
